# Caveolin-1 in renal cell carcinoma promotes tumour cell invasion, and in co-operation with pERK predicts metastases in patients with clinically confined disease

**DOI:** 10.1186/1479-5876-11-255

**Published:** 2013-10-11

**Authors:** Lee Campbell, Ghaith Al-Jayyoussi, Robert Gutteridge, Nigel Gumbleton, Rosie Griffiths, Simon Gumbleton, Mathew W Smith, David FR Griffiths, Mark Gumbleton

**Affiliations:** 1 Cardiff School of Pharmacy and Pharmaceutical Sciences, Cardiff University, Cardiff CF10 3XF, UK; 2 Department of Pathology, School of Medicine, Cardiff University, Heath Park, Cardiff CF14 4XN, UK

**Keywords:** Caveolin-1, ERK-1/2, Renal Cell Carcinoma, Invasion, Biomarkers

## Abstract

**Background:**

Up to 40% of patients initially diagnosed with clinically-confined renal cell carcinoma (RCC) and who undergo curative surgery will nevertheless relapse with metastatic disease (mRCC) associated with poor long term survival. The discovery of novel prognostic/predictive biomarkers and drug targets is needed and in this context the aim of the current study was to investigate a putative caveolin-1/ERK signalling axis in clinically confined RCC, and to examine in a panel of RCC cell lines the effects of caveolin-1 (Cav-1) on pathological processes (invasion and growth) and select signalling pathways.

**Methods:**

Using immunohistochemistry we assessed the expression of both Cav-1 and phosphorylated-ERK (pERK) in 176 patients with clinically confined RCC, their correlation with histological parameters and their impact upon disease-free survival. Using a panel of RCC cell lines we explored the functional effects of Cav-1 knockdown upon cell growth, cell invasion and VEGF-A secretion, as well Cav-1 regulation by cognate cell signalling pathways.

**Results:**

We found a significant correlation (*P* = 0.03) between Cav-1 and pERK in a cohort of patients with clinically confined disease which represented a prognostic biomarker combination (HR = 4.2) that effectively stratified patients into low, intermediate and high risk groups with respect to relapse, even if the patients’ tumours displayed low grade and/or low stage disease. In RCC cell lines Cav-1 knockdown unequivocally reduced cell invasive capacity while also displaying both pro-and anti-proliferative effects; targeted knockdown of Cav-1 also partially suppressed VEGF-A secretion in VHL-negative RCC cells. The actions of Cav-1 in the RCC cell lines appeared independent of both ERK and AKT/mTOR signalling pathways.

**Conclusion:**

The combined expression of Cav-1 and pERK serves as an independent biomarker signature with potential merit in RCC surveillance strategies able to predict those patients with clinically confined disease who will eventually relapse. In a panel of in-vitro RCC cells Cav-1 promotes cell invasion with variable effects on cell growth and VEGF-A secretion. Cav-1 has potential as a therapeutic target for the prevention and treatment of mRCC.

## Introduction

Caveolin-1 (Cav-1) is a regulator of signal transduction events and cytoskeletal dynamics [1,2]. In some cell types it interacts with multiple members of the EGF-R/RAS/ERK and PI3-K/AKT pathways to modify signalling activity [3]. At least in preclinical models Cav-1 is shown to modulate a number of signalling pathways to promote and/or suppress the malignant phenotype [4,5]. For example, Cav-1 has been shown to facilitate both ERK and AKT signalling in cancer cells derived from colon [6], prostate [7], epidermis [8] and smooth muscle [9], and is associated with promoting cell invasion, proliferation, angiogenesis and multi-drug resistance. However, the role of Cav-1 in malignancy is both complex and multifaceted with both tumour suppressor and oncogenic properties described in what appears to be a disease-specific and context-dependent manner. For example, the elevated levels of Cav-1 in clinical tumour tissue from prostate [10], bladder [11] and multiple myeloma [12] is unequivocally linked with metastasis and poor prognosis. Meanwhile in carcinomas of the breast [13,14], colon [15,16] and lung [17-19] both the loss and gain of Cav-1 have been associated with tumour progression.

Renal Cell Carcinoma (RCC) is a highly vascularised heterogeneous group of tumours with the clear cell phenotype the most common and aggressive form [20]. At diagnosis approximately one-third of RCC patients present with metastatic disease (mRCC) [21] which is highly resistant to conventional treatments and which is associated with a very poor long-term survival [22]. The mainstay of treatment for clinically confined RCC is curative radical nephrectomy, however, even in this group of patients upto 40% will eventually develop mRCC [22]. Identifying patients at high risk of relapse is compromised by the varying clinical course of patients whose primary tumours are of similar histological stage and grade [23] but which must display significant molecular heterogeneity. As such prognostication and post-operative patient surveillance with early instigation of molecular therapies would benefit from mechanistically-based biomarkers that accurately reflect the clinical significance of different RCC primary tumour biologies.

Previously, we and others have shown Cav-1 [24-26] to correlate with the aggressive features of RCC and predict poor disease-free survival (DFS) in patients presenting with clinically confined disease. We have also shown pERK-1/2 to be a significant predictor of poor DFS in RCC and shown it to serve as an independent prognostic biomarker [27]. We have also revealed co-operation between Cav-1 and the AKT/mTOR pathway in advanced RCC [28]. However, the importance and clinical significance of Cav-1 and pERK co-expression and co-operation is unknown and a full understanding of the roles of Cav-1 in RCC patho-biology remains to be determined. In this study we show a positive correlation in primary RCC tumours between the over-expression of Cav-1 and pERK-1/2, their co-expression in localised tumours a powerful biomarker combination able to stratify patients into low, intermediate and high risk of developing mRCC including recognising high risk patients whose primary tumours displayed low grade and/or low stage disease. We also found significant concordance in the expression of Cav-1 and pERK-1/2, either alone or combined, between matched primary and metastatic tumours. Consistent with pro-aggressive features of Cav-1 in the clinical data we show in a panel of RCC cell lines of varying genetic background that Cav-1 levels directly influence RCC cell growth and cell invasion, and its expression is associated with pro-angiogenic potential in VHL-negative RCC cells. However, under the same experimental conditions we found no direct control of either ERK upon Cav-1 expression or the reverse, i.e. Cav-1 upon ERK. Further, both the PI3-K/AKT/mTOR and the RANKL/NFkappB signalling modules, two important pathways in RCC, were also found to be without effect upon Cav-1 expression. These results corroborate Cav-1 to have direct effects on RCC patho-biology and support Cav-1 as a valuable biomarker in RCC especially when incorporated with other markers of biologically relevant signalling pathways such as activated ERK.

## Material and methods

### Human renal cell carcinoma cell lines and culture

Caki-1 and A498 cells were provided by Professor R.A. Blaheta (Johann Wolfgang Goethe University, Frankfurt) while 786-O and RCC4 cells were from Professor A. Harris (Radcliffe Hospital, Oxford). The caki-2 and ACHN cell lines were obtained from E.C.A.C.C. (Porton Down, UK). The caki-1, caki-2 and A498 cell lines were routinely cultured in RPMI medium (Invitrogen), while RCC4, 786-O, and ACHN cells were cultured in DMEM (Invitrogen). Both media were supplemented with 10% FBS and 1% penicillin G (100 units/ml) and streptomycin (100 μg/ml) and maintained in 5% CO_2_ at 37°C.

### siRNA treatments

Using methods previously described [29] a siRNA duplex (21 nucleotides) was used to down-regulate Cav-1 mRNA (siRNA sequence 5′-AGACGAGCUGAGCGAGAAGUU-3′) and a siRNA duplex (21 nucleotides) targeting the non-mammalian firefly luciferase (GL2) (siRNA sequence 5′-AACGUACGCGGAAUACUUCGA-3′) was used as a negative control. The duplexes were purchased from MWG (Ebersberg, Germany) as unprotected, desalted and purified siRNA.

For all transfection studies 786-O and A498 cells were seeded at a density of 1.3 × 10^3^ cells cm^-2^ and caki-1 cells at a density of 3 × 10^3^ cells cm^-2^, in either a 6-well format for Western blot and invasion studies or a 24-well format for growth assays. At 24 hrs post-seeding the cells were transfected (Oligofectamine, Invitrogen) with 50 pmoles siRNA targeting either Cav-1 or control (luciferase). Following a 4 hr transfection period the cells were supplemented with their respective culture medium containing 10% FBS. At 3 days (72 hrs) post-transfection (i.e. 98 hrs post-seeding) the cells were either collected for invasion studies, harvested for Western blot or evaluated for cell growth. Cell growth was assessed by MTT and definitively by means of trypsin dispersion of the cell monolayers with cell counts quantified by a Coulter-counter (Luton, UK). Despite several different transfection strategies an adequate and reproducible siRNA-mediated Cav-1 down-regulation was not achievable in RCC4 and ACHN cells.

### Cell treatments

For the pharmacological inhibitor studies the cells were seeded in 6-well and 24-well formats as described above. At 24 hrs post-seeding the cells were treated with either the mTOR inhibitor rapamycin (1 and 10 nM), the MEK inhibitor PD98059 (10, 25 and 50 μm) or the PI3-K/AKt inhibitor LY-294002 (10 and 50 μm); cells were incubated in the drug of choice for 48 hrs or 72 hrs. Cells were used for growth assay or harvested for immunoblot. For the RANK-L studies cells were grown in a 6-well format for 48 hrs in the presence of serum at which point they were serum starved overnight (16 hrs). After this RANK-L(100 ng/ml) was added and the cells then harvested at 24 hrs post-treatment for immunoblot.

### Immunoblotting

Cells were seeded in a 6-well format as described above and treated with either siRNA or the drug of choice. At the indicated times post-treatment (24 to 72 hrs) cells were lysed (15 min on ice) using ice-cold lysis buffer, then centrifuged at 12,000 g for 15 min at 4°C. Total protein concentrations were determined using the BC BioRad protein assay kit (Bio-Rad, Hertfordshire, UK). Cell lysates of equivalent total protein were denatured and resolved on 12% SDS polyacrylamide gels and electro-blotted onto 0.2  μm nitro-cellulose membrane (Schleicher and Schuell, Dassel, Germany). Membranes were blocked (1 hr) with blocking buffer consisting of 5% fat-free dry milk in Tris buffered saline (TBS)-Tween 20 (0.05%) (TBS-T; pH 7.5) and then incubated with the primary antibody of choice for 16 hrs at 4 °C (1:1000 in blocking buffer). All primary antibodies were from Cell Signalling (New England Biolabs, Hertfordshire, UK) unless otherwise stated: Cav-1, phospho-AKT (Ser473), total AKT, phospho-S6 (Ser235/236), total S6, phospho-ERK-1/2 (Tyr42/44), total ERK, phospho-NF-KappaB-p65 (Ser536), total NK-KappaB-p65, c-myc and β-actin. Cyclin D1 and α-tubulin were from Santa Cruz (USA). After primary antibody incubation the membranes were washed (6 × 5 min) in TSB-T and then incubated for 1 hr at room temperature with the required secondary IgG HRP-labelled antibody (Cell Signalling) diluted 1/7000 in blocking buffer. Membranes were again washed (6 × 5 min) in TSB-T and signals detected using either SuperSignal™ WEST DURA or FEMTO chemiluminescent substrate (Pierce, Chester, UK). Signals were captured on ECL Hyperfilm, following between 1 and 30 min exposures and the film developed and fixed as appropriate.

### Renal cancer cell invasion assays

To evaluate the effects of Cav-1 silencing on RCC cell invasion, cells were transfected with either anti-Cav-1 or control siRNA as described above. The invasion assays were conducted using Transwell^TM^ cell culture inserts (6.5 mm diameter, 8 μm pore size; Costar, supplied by Fisher, UK). The upper surface of the inserts were coated with Matrigel™ (0.4 μg/ml) in a sterile tissue-culture hood and allowed to polymerise at 37°C for 2 hrs prior to use. Cells treated with anti-Cav-1 or control siRNA were trypsinised (72 hrs post-transfection) from the 6-well culture dishes and 5 × 10^4^ cells re-suspended in 200 μl of culture media and seeded into the upper chamber of the coated inserts with 650 μl of culture media added to the bottom chamber. Cell invasion was allowed to progress at 37°C for 24 hrs after which the non-invasive cells and Matrigel™ were gently removed from the upper surface of the polycarbonate membrane using a cotton swab. Cells that had invaded to the lower surface of the membranes were fixed with 3.7% formaldehyde and counterstained with 0.05% Hoeschst 33258 in PBS (pH 7.4) for visualisation of nuclei. The Transwell membranes were then removed from the insert and mounted onto microscope slides (Vectashield™, Molecular Probes). Cell invasion was quantified by photographing the membranes and selecting five random fields of view at x20 magnification. Cells were counted and data plotted as a percentage of control for a minimum of three independent experiments (each performed in duplicate).

### VEGF-A ELISA assay

For determination of VEGF-A secretion following Cav-1 knockdown, cells were cultured in a 6-well format and treated with either anti-Cav-1 or control siRNA. At 72 hrs post-transfection the cell culture supernatants were collected and the secreted levels of VEGF-A quantified (Quantikine human VEGF-A ELISA kit; R&D Systems). Cells were harvested using lysis buffer and the total VEGF-A levels present in the supernatants was normalised to total cellular protein levels.

### Patient cohorts and tissue microarray construction

This study was approved by the South East Wales Research Ethics Committee. Two separate tissue microarrays (TMA) were constructed. One consisted of 174 biopsy or radical nephrectomy samples resected from patients with clinically confined RCC as described in detail elsewhere [27,28]. The other TMA consisted of a cohort of matched primary and metastatic tumour specimens from 14 patients who had undergone surgery for removal of both primary and secondary tumours either simultaneously or at a later date. In all of the above cases archival paraffin-embedded tissue blocks, histology reports and slides were available and for each tissue specimen used in the construction of the TMA a block was selected that contained a sample of peripheral tumour.

Construction of TMAs and immunohistochemistry for Cav-1 and pERK-1/2 was undertaken using previously published methodologies [27,28]. Briefly, single cores representative of peripheral tumour (0.6 mm in diameter) were punched from each donor block and transplanted into a pre-moulded recipient paraffin wax block. Additional 'control’ cores were taken from normal renal tissue (adjacent to some of the tumours) and from human placenta. Serial sections were cut (4 μm thickness) from the resulting TMA block and placed onto cleaned adhesive glass slides (Superfrost Plus™).

### Immunohistochemistry and scoring of sections

Array sections were deparaffinised and rehydrated using 3 sequential changes of 100% xylene and 100% ethanol, respectively. Antigen retrieval for pERK-1/2 and Cav-1 was undertaken as previously described [24,27,28]. Briefly, following removal of the paraffin wax the endogenous peroxidase activity within the rehydrated tissue was quenched using 3% hydrogen peroxide for 5 min. For pERK-1/2, antigen retrieval consisted of microwaving TMA sections in citric acid (0.1 M, pH 6.0) for 30 min, while for Cav-1 antigen retrieval consisted of boiling slides in citric acid for 20 min. In all cases slides were cooled with running tap water and after draining the array sections were equilibrated (15 min at room temp) in either 100% normal human serum (pERK-1/2), or 0.6% BSA in Optimax™ wash buffer (Cav-1). Primary rabbit anti-human pERK-1/2 and Cav-1 antibodies were applied to each section at a dilution of 1:25 and incubated for 16 hrs at 4°C. The next day sections were washed (4 × 1 min) with PBS and tissue immunostained using the DAKO rabbit Envision™ staining system (DAKO, Cambridge, UK) according to the manufacturers instructions. The TMA sections were counterstained with haematoxylin and finally mounted.

Tumour arrays were scored by a pathologist (DFRG) and team members (LC and MG) without knowledge of other pathological and clinical data. Expression of both Cav-1 and pERK-1/2 was assessed using a semi-quantitative criteria as previously described [24,28] that accounted for both the intensity of immunostain within tumour cells and the percentage of tumour cells involved in each core. Scoring was as follows: 0: no detectable immunostain in tumour cells; 1: very light diffuse or focal light staining in tumour cells; 2: light diffuse or moderate focal staining (may include very small areas of heavy deposit); 3: tumour cores containing areas of heavy staining in most or all tumour cells. The scores were also converted to a simple binary positive or negative score.

### Statistical analysis

#### ***Statistical analysis of clinical data***

Kaplan-Meier survival analysis was conducted to calculate the disease-free survival (DFS) of patients with tumours showing different scores for Cav-1 and pERK-1/2 staining. This method was carried out using the log-rank test where the first appearance of a metastasis was considered an event and with patients considered censored who were last seen alive without metastasis or who had died due to other causes. Scores were converted to a binary simple covariate (positive or negative) by thresholding to the most informative split on the Kaplan-Meier using the log-rank statistical test. For Cav-1 a score of 0 and 1 was negative, and a score of 2 and 3 was positive, while for pERK-1/2 the presence of any staining was considered positive. To test for synergy between Cav-1 expression and pERK-1/2, composite covariates were constructed and considered positive if both Cav-1 and pERK-1/2 were expressed in the same patient tumour and negative if expression of either of these markers were negative. The association of biomarker expression with conventional histological parameters (grade, size, vascular invasion, stage, tumour type and capsular invasion) was examined by cross tabulation and the chi-squared test.

Multivariate survival analysis was carried out by Cox regression using the “Enter or Forward Stepwise (Likelihood)” function with covariates considered categorical. We had already determined that the most influential covariates predicting disease-free progression of these patients are Fuhrman grade (grades 1 and 2, and grades 3 and 4 are pooled for analysis), any degree of vascular invasion, tumour stage and histological evidence of renal capsular invasion. When these covariates are taken into account then tumour size and type had no influence on DFS [30]. To determine if any of the biomarker covariates had predictive value in the multivariate analysis, each covariate (both simple and composite) was added individually in turn as an independent covariate to the Cox regression analysis together with tumour grade, stage, vascular invasion and invasion of the renal capsule; time to event being the dependent variable.

To assess the concordance of both Cav-1 and pERK between primary and metastatic tumours the IHC staining on the tissue microarray cores were classified into four categories according to the IHC phenotype of each core (Cav+, pERK+; Cav-, pERK-; Cav+, pERK-; Cav-, pERK+) with the presence of any staining considered positive. The results from primary and metastatic tumours were cross tabulated and the concordance assessed using the Pearson contingency coefficient for paired observations and Kappa statistic.

*Statistical analysis of preclinical data* Preclinical data was analysed for two groups by T-test (unpaired) and by more than two groups using ANOVA with post-hoc tests Dunnett (comparisons to control) or Duncan (comparisons across all groups). Statistical significance at P ≤ 0.05.

## Results

### Combined Cav-1 and pERK-1/2 expression in localised RCC tumours is a powerful predictor of metastasis

**Figure 1:**
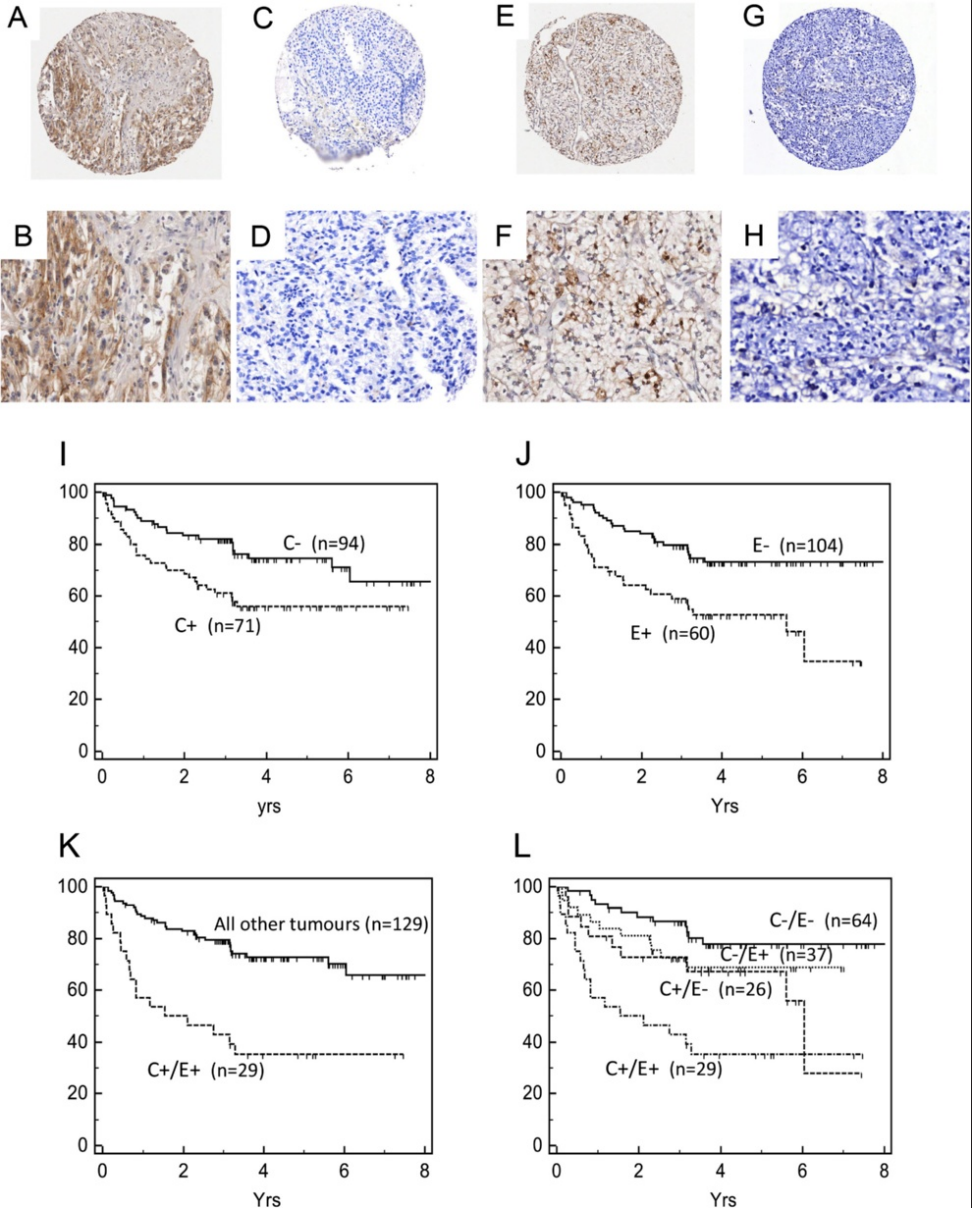
**Correlation of Cav-1 and pERK-1/2 expression in clinically confined RCC.** Representative microarray cores of Cav-1 **(1A** to **1D)** and pERK-1/2 **(1E** to **1H)** demonstrating positive and negative immunohistochemical staining in RCC. Typical cores are shown in low **(1A**,**1C**,**1E**,**1G)** and high power **(1B**,**1D**,**1F**,**1H)**. Kaplan-Meier metastasis-free survival of RCC patients (%) presenting with clinically confined disease whose primary tumours were stratified by positive (+) or negative (-) expression of: Cav-1 **(1I)**, pERK-1/2 **(1J)**, co-expression of pERK-1/2 and Cav-1 [C+/E+] **(1K)**, and sub-analysis **(1L)** of patients who were: negative for both Cav-1 and pERK-1/2 [C-/E-]; positive for pERK-1/2 alone [C-/E+]; positive for Cav-1 alone [C+/E-]; positive for both biomarkers [C+/E+]. Patients whose tumours expressed both pERK-1/2 and Cav-1.showed a significantly worse prognosis. Note: Figures **1I** and **1J** relate to the univariate data for Cav-1 and p-ERK separately while Figures **1K** and **1L** relate to the co-variate data for Cav-1 and p-ERK combined (Table 2).

In clinically confined RCC we investigated the correlation between Cav-1 and pERK-1/2 levels in primary tumours and sought to examine if their combined expression provided an enhanced prognostic indicator. Of the samples that could be analysed for the combined expression of Cav-1 and pERK-1/2 we found (Table 1) 42% (66/158) of patient tumours were positive for Cav-1 (Figure 1A and 1B) and 35% (55/158) positive for pERK-1/2 (Figure 1E and 1F). Cross tabulation revealed the levels of Cav-1 and pERK-1/2 to be significantly (*P* = 0.03) associated in the primary RCC tumours with 19% (29/158) of tumours showing co-expression. We found the association between Cav-1 and pERK-1/2 only in the clear cell tumours, the most aggressive RCC histological subtype. In the more indolent papillary carcinomas only 1 from 20 papillary tumours examined were positive for both biomarkers (Table 2). The combined expression of Cav-1 and pERK-1/2 correlated with clear cell histology, high tumour stage and vascular invasion (Table 2). Kaplan-Meier survival analysis revealed patients with Cav-1 positive tumours had a mean DFS of 4.72 yrs versus 6.35 yrs (*P* = 0.013) for patients with Cav-1 negative tumours (Figure 1I). Patients with pERK-1/2 positive tumours had a DFS of 4.19 yrs versus 6.38 yrs (*P* = 0.001) for pERK-1/2 negative tumours (Figure 1J). Notably, patients whose tumours simultaneously expressed Cav-1 and pERK-1/2 had a DFS of only 3.33 yrs versus 6.17 yrs (*P* = 0.001) for tumours either negative for one or both of the biomarkers (Figure 1K).

**Table 1:** Correlation of Cav-1 expression with pERK in clinically confined RCC

**p-ERK score**	**Cav-1 negative**	**Cav-1 positive**
0	66	37
1	12	13
2	7	14
3	7	2

Statistically significant correlation *P* = 0.03.

**Table 2:** Association of the combined expression of Cav-1 and pERK with conventional histological parameters in RCC

	**Cav-1 (n = 165)**		**p-ERK1/2 (n = 164)**		**Cav-1/pERK1/2 (n = 158)**	
	-	+	-	+	All other Tumours	+/+
Grade 1 & 2	69	47	75	43	93	19
Grade 3 & 4	25	24	29	17	36	10
		*P* = 0.390		*P* = 0.951		*P* = 0.091
Tumour size <7 cm	49	29	55	24	64	10
>7 cm	45	42	49	36	65	19
		*P* = 0.151		*P* = 0.112		*P* = 0.142
Vascular Invasion (-ve)	66	33	71	30	85	10
(+ve)	28	38	33	30	44	19
		*P* = 0.002*		*P* = 0.021*		*P* = 0.002*
Capsular Invasion (-ve)	83	55	90	47	109	22
(+ve)	11	16	14	13	20	7
		*P* = 0.063		*P* = 0.172		*P* = 0.267
Tumour Stage 1	44	21	50	17	55	7
Stage 2	31	14	28	15	38	4
Stage 3 & 4	19	36	26	28	36	18
		*P* = 0.0001*		*P* = 0.011*		*P* = 0.002*
Non- papillary	76	67	84	58	109	28
Papillary	18	4	20	2	20	1
		*P* = 0.011*		*P* = 0.004*		*P* = 0.084

* Indicates that statistical significance achieved to at least P < 0.05 with actual P value indicated in the Table itself.

Stratifying patients into four subgroups according to the expression of tumour Cav-1 and pERK-1/2 (Figure 1L) revealed that tumours lacking expression of both Cav-1 and pERK-1/2 (C-/E- n = 64) were associated with a good prognosis, i.e. DFS of 6.75 yrs and an 80% probability of remaining disease-free at 5 yrs. This compared to a DFS: of 5.27 yrs (*P* = 0.001) where tumours were Cav-1 positive and pERK-1/2 negative (C+/E- n = 26); of 4.77 yrs (*P* = 0.001) where tumours were pERK-1/2 positive and Cav-1 negative (C-/E + n = 37), and of 3.33 yrs (*P* = 0.001) where tumours co-expressed pERK-1/2 and Cav-1 (C+/E + n = 29). Notable was that the combined covariate of Cav-1 and pERK-1/2 was able to identify patients at high risk of relapse even if the patients’ tumour displayed low grade (19/29 patients positive for the co-variate and relapsing early had grade 1 or 2 tumours) and/or low stage (11/29 had stage 1 or 2 tumours) disease. Multivariate Cox regression analysis (Table 3) found only tumour grade (HR = 3.4; *P* = 0.001), capsular invasion (HR = 5.4; *P* < 0.001) and combined expression of Cav-1 and pERK-1/2 (HR = 4.2; *P* < 0.001) showed significant association with reduced DFS. For Cav-1 alone the HR =1.5 (95% CI 0.83-2.55; *P* = 0.18) while for pERK-1/2 alone the HR = 2.96 (95% CI 1.77-5.35; *P* = 0.001).

**Table 3:** Multivariate Cox regression hazard model for time to recurrence using a forward conditional selection method

**Prognostic indices model (n)**	**HR**	**95% CI**	***P *** **value**
Grade 1 and 2	1		
Grade 3 and 4	3.4	1.9-6.1	<0.001*
No capsular invasion present	1		
Capsular invasion present	5.4	3.0-10.0	<0.001*
Cav-1 and p-ERK1/2 covariate negative	1		
Cav-1 and p-ERK1/2 covariate positive	4.2	2.3-7.5	<0.001*

Covariates used include: tumour grade, stage, size, capsular invasion, vascular invasion, Cav-1 and p-ERK1/2. The covariates of size, stage and vascular invasion are rejected as not influential in the model by the selection method.

CI = 95% confidence limit, HR = Hazard Ratio *denotes significance *P* < 0.001.

### Concordance of Cav-1 and pERK-1/2 expression between paired primary and metastatic tumours

**Figure 2:**
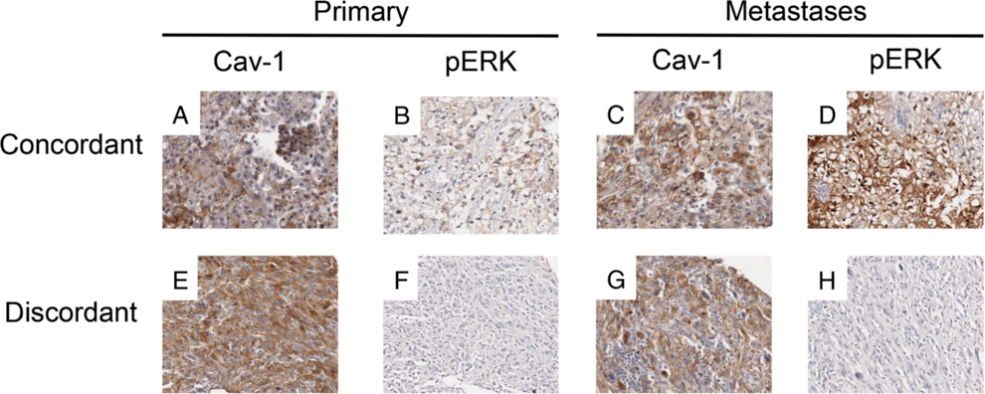
**Concordance of Cav-1 and pERK-1/2 between matched primary and secondary.** Typical immunohistochemical staining in matched primary and secondary tumours showing (i) positive concordance of both Cav-1 and pERK-1/2 **(2A**-**2D)** and (ii) discordance of Cav-1 and pERK-1/2 **(2E**-**2H)**.

Concordance of Cav-1 and pERK-1/2 expression was assessed for 16 available matched primary and secondary (mRCC) tumours obtained from 14 different patients (Figure 2A to 2H; Table 4); in two patients mRCC tissue was available from two sites. Six metastases were identified synchronously with the primary (4 lymph nodes; 2 adrenal), the others between 5 months and 10 years after nephrectomy (2 liver, 2 bone, 2 brain, 2 adrenal, 1 lung, 1 soft tissue). In 14 of 16 pairs Cav-1 expression was concordant (88%) between primary and metastatic tumours. In the two discordant cases Cav-1 was expressed in the primary tumour but not in the secondary. For pERK-1/2 a 94% level of concordance was observed, with the one discordant case showing a lack of pERK-1/2 in the secondary tumour but a presence in the primary. For the combined covariate of Cav-1/pERK-1/2, the primary and secondary tumours pairs were 88% (14/16) concordant. For all three cross-tabulations the agreement between primary and metastatic tumours was significant (P < 0.01; Pearson contingency coefficient) with Kappa values 0.64 to 0.74 indicating good to substantial agreement.

**Table 4:** Concordance of Cav-1 and pERK expression in matched primary and secondary metastatic tumours

**Primary**	**Metastatic**				**Concordance**		
Biomarker	Cav (-)	Cav (+)	pERK (-)	pERK (+)	Concordant / Total	Kappa	P
Cav (-)	3	0			14/16	0.67	P = 0.004
Cav (+)	2	11					
pERK (-)			1	0	15/16	0.64	P = 0.006
pERK (+)			1	14			
	Cav(-) pERK(-)	Cav(+) pERK(-)	Cav(-) pERK(+)	Cav(+) pERK(+)			
Cav(-)/pERK(-)	0	0	0	0	14/16	0.74	P < 0.001
Cav(+)/pERK(-)	0	1	0	0			
Cav(-)/pERK(+)	0	0	3	0			
Cav(+)/pERK(+)	1	0	1	10			

### Cav-1 is expressed in both VHL-negative and VHL-positive RCC cell lines where it modulates growth and drives invasion

**Figure 3:**
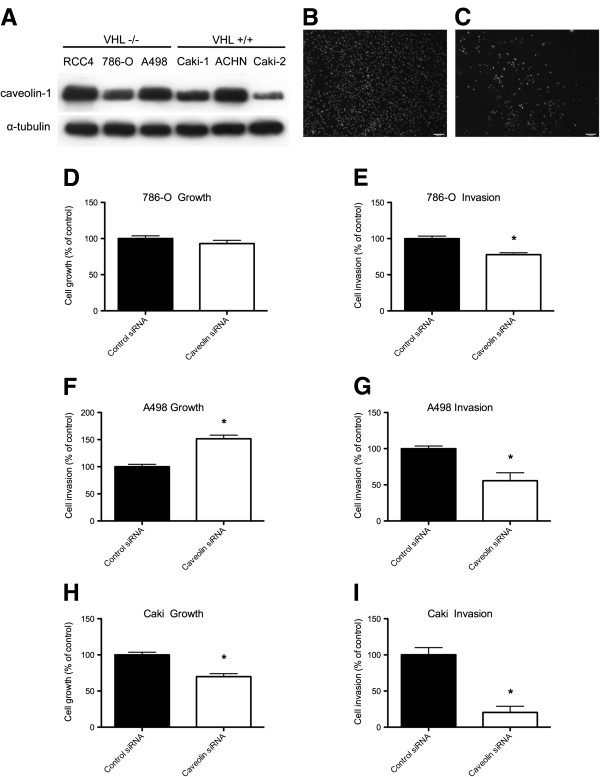
**In-vitro Growth and invasion of RCC cell lines. (3A)** - Cav-1 is expressed in both VHL negative and VHL positive RCC cell lines under normoxic conditions Western blot showing comparative levels of Cav-1 protein (20 μg) expression in a panel of RCC cell lines. Blot is representative of at least 3 independent experiments; **(3B** and **3C)** - Typical fields of view, respectively, for control siRNA or anti-Cav-1 siRNA treated caki-1 cells invading Matrigel coated membranes for 24 hrs and counterstained with Hoeschst 33258. Coated membranes were set up in duplicate with the data represents the SEM of 3 different experiments; **(3D**, **3F**, **3H)** - Effect of Cav-1 silencing on RCC cell growth (mean ± SD, n = 6) and **(3E**, **3G**, **3I)** - Effect of Cav-1 silencing on RCC cell invasion through Matrigel.

It has been previously reported [31] that Cav-1 expression in the RCC cell line 786-O is regulated by VHL- and Hif-dependent mechanisms. Here, at least under normoxic conditions we found Cav-1 protein to be ubiquitously expressed in a panel of primary and metastatic RCC cell lines (Figure 3A) independent of VHL-status and indeed Hif expression, for example, the ACHN (VHL-positive) cell line expresses negligible Hif under normoxic conditions [32,33].

**Figure 4:**
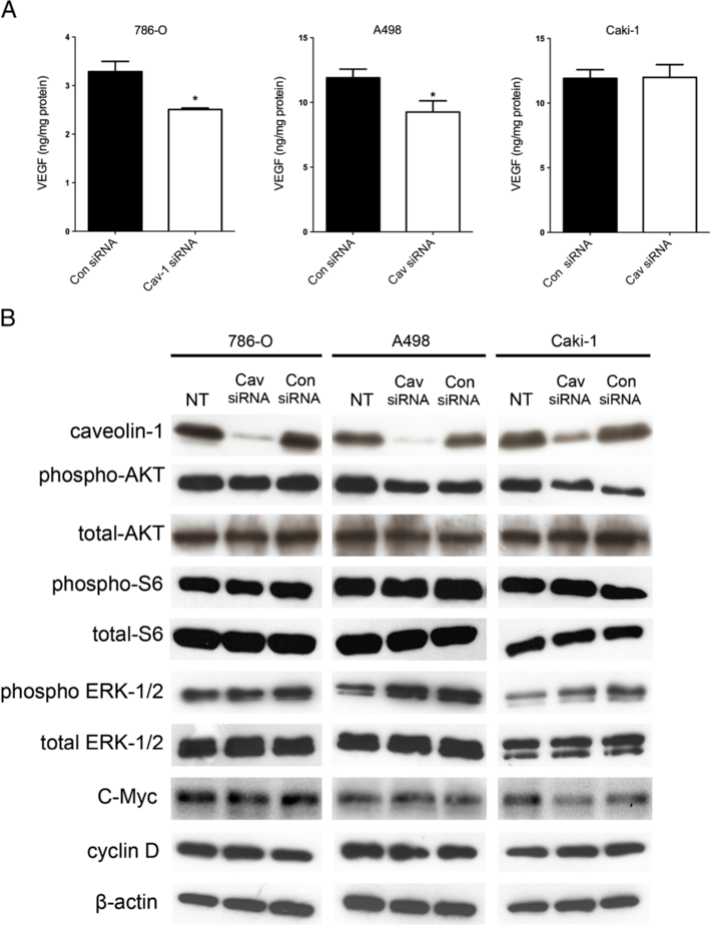
**Effect of Cav-1 knockdown on signalling molecules in RCC cell lines. (4A)** - Secretion of VEGF-A in RCC cells. Data is expressed as mean ± SD (n = 3) and is representative of two independent experiments; **(4B)** - Activity of AKT/mTOR and ERK signalling pathways and expression of the cell cycle regulators cyclinD1 and c-myc in RCC cell lines as a function of Cav-1 siRNA-mediated knockdown. Western blot is representative of at least four independent experiments.

We explored the role of Cav-1 in RCC tumorigenic potential through in-vitro studies in the 786-O (VHL-/- and PTEN-/-), A498 (VHL-/- and PTEN+/+) and caki-1 (VHL+/+ and PTEN+/+) cell lines all of which are of clear cell origin. Treatment with anti-Cav-1 siRNAs consistently resulted in a substantial reduction of Cav-1 protein (Figure 4B). The knockdown in Cav-1 had varying effects on cell proliferation: no effect in 786-O cells (Figure 3D), increases (50%, P < 0.001) in A498 proliferation (Figure 3F) and decreases (30%, P < 0.001) in caki-1 proliferation (Figure 3H). In contrast, silencing of Cav-1 consistently reduced (P < 0.001) cell invasiveness by 25% in the 786-O (Figure 3E), by 40% in A498 (Figure 3G) and 70% in caki-1 (Figure 3I); typical fields of view for Matrigel invasion by caki-1 cells are shown in Figure 3B (control siRNA) and Figure 3C (anti-Cav-1 siRNA). While the effects of Cav-1 upon RCC cell proliferation were cell line dependent we found an unequivocal role for Cav-1 in promoting RCC cell invasion.

### Cav-1 down-regulation in RCC cells and effects on AKT/mTOR and ERK signalling, and VEGF-A secretion

Cav-1 siRNA down-regulation resulted in an approximate 25% reduction (*P* = 0.05) in VEGF-A secretion (Figure 4A) in the VHL-negative 786-O and A498 RCC cell lines, while no significant effect upon VEGF-A secretion was seen in the VHL-positive caki-1 cells. As such Cav-1 appears to have a partial role in mediating the secretion of VEGF-A in RCC cell types that maybe dependent on VHL status. Substantial siRNA-mediated suppression of endogenous Cav-1 protein expression did not, however, have any noticeable effect on the basal levels of phosphorylated AKT, phosphorylated ERK and phosphorylated S6 (Figure 4B), at least under non-stressed conditions, indicating that alterations to AKT/mTOR and ERK signalling are not implicated in the observed effects of Cav-1 down-regulation upon cell growth, invasion and VEGF-A secretion. The levels of the pro-proliferative cell cycle regulators, cyclin D1 and c-myc, also remained relatively unchanged in all three RCC cell lines.

### AKT/mTOR and ERK down-regulation and RANKL stimulation in RCC cells and effects on Cav-1 expression

**Figure 5:**
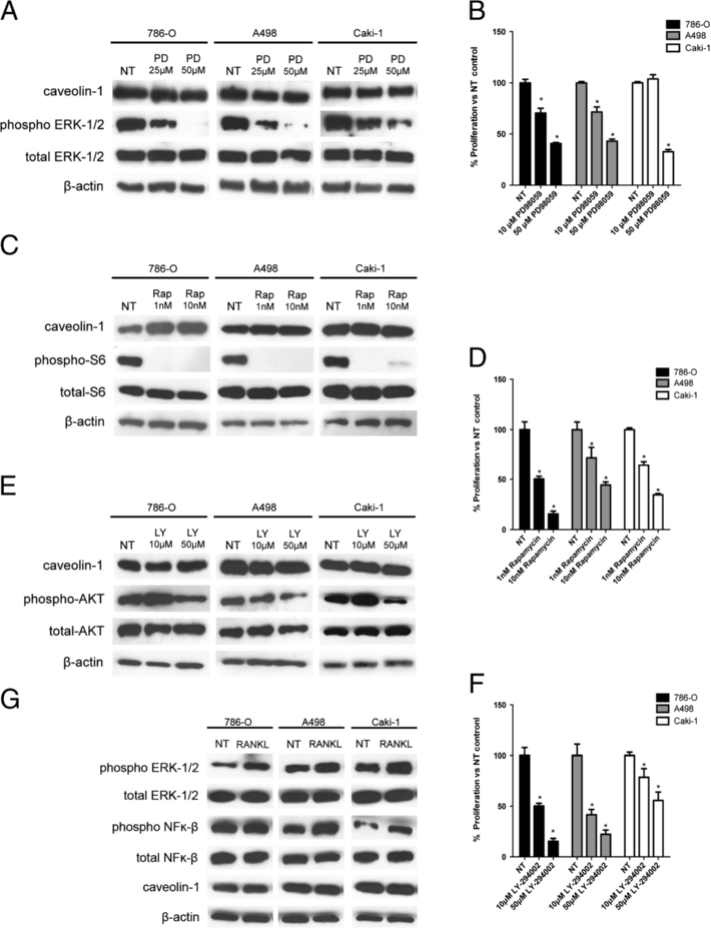
**Pharmacological knockdown of signalling molecules and effect upon Cav-1 in RCC cell lines. (5A and 5B)** - Effects of inhibiting ERK upon Cav-1 and cell growth; **(5C and 5D)** - Effects of inhibiting TORC1 upon Cav-1 and cell growth; **(5E and 5F)** - Effects of inhibiting PI3-AKT upon Cav-1 and cell growth. **(5G)** - Effect of RANKL on the stimulation of ERK and NF-kappaB pathways and Cav-1 expression. All above data represent mean ± SD (n = 6) and are representative of 3 independent studies.

In all three RCC cell lines the selective ERK inhibitor PD98059 (treatment 72 hrs) led to dose-dependent reductions in pERK-1/2 (Figure 5A) and decreases in cell proliferation (Figure 5B) but had no effect upon Cav-1 expression (Figure 5A), indicating Cav-1 is not serving as an immediate downstream effector molecule of ERK-1/2. Inhibition of mTORC1 with rapamycin (48 hr) resulted in profound loss of pS6 (Figure 5C) and a significant reduction in cell proliferation (Figure 5D) with cell-type dependent effects upon Cav-1, i.e. mTORC1 inhibition caused a significant increase in the expression of Cav-1 protein in the PTEN-negative 786-O cells but no change in either of the PTEN-positive cell lines, A498 and caki-1. Treatment with the PI3-K inhibitor, LY-294002 (48 hrs), resulted in inhibition in AKT signalling (Figure 5E) and reductions in cell proliferation (Figure 5F), but was without effect upon Cav-1 expression (Figure 5E).

RANKL is a member of the TNF superfamily and triggers multiple signalling pathways. It has been associated with tumour migration and metastasis in clinical cases of RCC and invasion in in-vitro experiments with caki-1 cells [34]. Following RANKL stimulation we observed increased expression of phosphorylated ERK in all three RCC cell lines accompanied by increased phosphorylated NF-kappaB in A498 and caki-1 cells (Figure 5G). However, no change in Cav-1 protein expression was observed in any of the three RCC cell lines (Figure 5G) implying that NF-kappaB does not serve as an immediate upstream effector for Cav-1 ,at least in this experimental setting.

## Discussion

The high relapse rates for patients diagnosed with clinically confined disease, the treatment resistant nature of mRCC, and the potential benefits of new molecular therapies would lend support for improved measures to identify patients at high risk [21,35]. At present tumour grade and stage are the standard determinants used in RCC to predict disease recurrence, although both have limitations [23].

In this current study we show in primary RCC tumours correlation between the increased expression of pERK-1/2 and Cav-1, and that their combined expression serves as a more powerful predictor of disease recurrence than tumour stage or pERK-1/2 or Cav-1 alone. Thus Cav-1 and pERK-1/2 appear to co-operate imparting a growth and survival advantage to facilitate metastatic spread and early relapse. The combined covariate of Cav-1 and pERK-1/2 reliably stratified patients into low, intermediate or high risk of relapse including patients that display low grade and/or low stage disease. Further, we found biomarker concordance between matched primary and secondary tumour sites which supports similarities in respective tumour biology and which may allow primary tumour characterics to direct the choice of molecularly targeted therapies in mRCC [36,37]. Collectively our clinical findings would appear to have importance in the identification of high-risk clear cell RCC patients, and potentially subsequent instigation of treatment with molecularly targeted therapies to prevent or delay disease recurrence, or indeed in the use of such therapies in the treatment of mRCC itself. We did not identify an association between Cav-1 and pERK in papillary RCC tumours which contrasts to the report of Wang et al. [31] who found Cav-1 and pERK-1/2 co-expression in 100% (8/8) of their papillary RCC patient cases. However the latter data was generated by immunoblotting methods which lack the spatial co-localisation of protein stain to the tumour cells, a feature inherent in our use of immunohistochemistry methodologies.

We showed a high proportion of metastatic tumours to be Cav-1 positive (69%), a result consistent with the previous small case study of Hayakawa and co-workers [25] reporting Cav-1 expression in 83% (5/6) of secondary tumours. Our clinical data revealed (Table 2) a statistically significant association between Cav-1 expression and vascular invasion (*P* = 0.002, n = 165) and a strong trend in the relationship between Cav-1 expression and capsular invasion (*P* = 0.06, n = 165). We therefore undertook a series of in-vitro invasion assays to examine directly if Cav-1 represents a pro-metastatic gene. Using three human RCC cell lines derived from tumours of clear cell origin we found Cav-1 promoted invasiveness in all the models examined irrespective of genetic background of the cell line. This is the first report to show that Cav-1 is an important and direct mediator of invasion in *bone fide* human RCC cells of clear cell origin; although Cav-1 status has previously been reported not to influence the invasion of SN12CPM6 cells [38] (a RCC cell line of mixed histology). Very recently Yamasaki et al. [39] reported silencing of caveolin-2 (Cav-2; another caveolin family member) in 786-O and A498 cells reduced cell invasion and growth. While this is intriguing these workers did not evaluate the role of Cav-2 as prognostic biomarker in RCC nor did they elude to the potential co-dependency upon Cav-1. For example, intact caveolae are present in the 786-O cells [31,40] with these structures appearing to be involved in NEU3-mediated cell invasion through the regulation of β1 integrin endocytosis [40]. While Cav-1 drives the assembly of caveolae Cav-2 can in some circumstances regulate the size and shape of caveolae [41]. Further, the exact scaffolding domain present within the Cav-1 molecule that is known to interact and regulate multiple signalling molecules is absent in Cav-2 [2].

Although our in-vitro studies unequivocally support a role for Cav-1 in RCC invasion the impact of Cav-1 upon cell proliferation was more variable. It is however recognised that a signal regulatory molecule can display a dichotomy of control, for example inhibiting proliferation in favour of increased invasion and survival in cancer cells [42], e.g. YB-1 in breast cancer [43] and spastin [44] in glioma, opposing properties confering a survival advantage during the development of micrometastasis [42]. Our current studies in caki-1 cells (mRCC origin) show Cav-1 to be both pro-proliferative and pro-invasive, and may reflect the greater reliance of advanced and metastatic RCC tumours upon Cav-1 for their patho-biology.

RCC is a highly vascular tumour and previous studies have shown a significant positive correlation between tumour Cav-1 levels and high microvessel density [26]. We show in our in-vitro studies Cav-1 to have a partial role in mediating the secretion of VEGF-A. Specifically, under normoxic conditions Cav-1 elevated the secretion of the VEGF-A from the VHL-negative 786-O and A498 cells although not from the VHL-competent caki-1 cells. These differences may reflect the VHL status of the cells and/or the Hif isoforms the cells’ constitutively express. For example, Hif-2α is the major Hif isoform present in 786-O and A498 cells, known to be responsible for VEGF-A production and secretion, while Hif-2α appears to absent under normoxic in the VHL-positive caki-1 cells ([33] and our own observations). The AKT/mTOR pathway itself has been implicated in the regulating the expression of several key pro-angiogenic factors such as Hif [45] and VEGF [46]. Here we found the Cav-1 mediated increases in VEGF-A secretion to be independent of PI3-K/AKT and mTOR signalling, whereas Cav-1 appears to promote both the production and release of VEGF-A [47] in prostate cancer cells at least in part through the potentiation of PI3-K/AKT signalling.

Using in-vitro RCC models we investigated the relationship between Cav-1 expression and other related cell signalling pathways. Increased ERK-1/2 signalling can promote the expression of Cav-1 in various human cancer cell lines including those derived from the prostate [7] and smooth muscle [9]. In the current studies pharmacological inhibition of ERK signalling in the RCC cell lines did not affect Cav-1 protein expression. This suggests the positive correlation seen between Cav-1 and pERK-1/2 in the clinical samples is not a result of Cav-1 serving as an immediate downstream effector molecule of ERK-1/2 signalling. Our in-vitro studies in the RCC cell lines also show pERK to be maintained in the presence of Cav-1 down-regulation. This observation is consistent with Wang et al., [31] reporting Cav-1 to not effect the constitutive activity (i.e. in the presence of ligand/serum) of ERK signalling in the RCC line, 786-O. These authors did however find Cav-1 was able to maintain levels of pERK-1/2 under serum-deplete (ligand-independent) conditions.

We found inhibition of mTOR (TORC1) signalling to significantly increase Cav-1 expression in the PTEN-negative 786-O cells, but not in either of the PTEN-positive A498 or caki-1 cell lines. The basis for this is unclear, however, rapamycin is known to induce oxidative stress in cells [48] which is exacerbated by PTEN deletion [49]. Several oxidative stress elements that can serve a transactivation function are contained within the Cav-1 promoter [50]. RANKL is a common upstream effector of both ERK and NF-kappaB signalling that has been linked with metastasis in RCC, and with cell migration in in-vitro studies with caki-1 cells [34]. We examined in the in-vitro RCC models if RANKL served as a common upstream yet parallel effector of both pERK and Cav-1. While we saw increased activity of both ERK and NF-kappaB signalling following RANKL treatment, the levels of Cav-1 protein remained unchanged. This indicates that expression of Cav-1 in the in-vitro RCC cells was not maintained through enhanced NF-kappaB signalling, and that the functional properties of Cav-1 in the in-vitro assays were not downstream of RANKL-dependent pathways.

In summary, we demonstrate in clinically confined RCC tumours that Cav-1 expression when combined with the functionally relevant signalling molecule, pERK-1/2, provides a powerful prognostic biomarker able to stratify patients into low, intermediate and high-risk of metastatic relapse, a discovery potentially useful in guiding stratification in clinical trials and therapy. We report a significant concordance in the expression of Cav-1 and pERK-1/2 between primary tumours and matched metastatic tissue which supports the use of localised tumour biology to guide therapy of non-resectable mRCC. In a panel of RCC cell lines we provide for the first time unequivocal direct evidence that Cav-1 can directly promote the invasion of RCC-cell lines. We also show that Cav-1 stimulates pro-angiogenic signals in RCC cells through its ability to enhance secretion of VEGF-A. The in-vitro assays showed Cav-1 expression to be independent of ERK and AKT/mTOR signalling. The data presented here indicate that Cav-1 is an important biomarker and metastatic gene. The targeting of Cav-1 may represent a future strategy for the prevention and treatment of metastases or even micrometastasis before the development of overt secondary tumours.

## Competing interests

The authors declare that they have no competing interests.

## Authors’ contributions

MG, LC and DG contributed to the study concept, design, drafting of the manuscript and critical review of the final version. LC undertook the immunohistochemistry and with GJ conducted the invasion and growth studies. GJ and RG conducted the VEGF-A assays. RG, MS, SG, NG, RG and LC all contributed to the drug, growth and Western blot studies and interpretation of data. DG sourced human specimens, guided immunohistochemical assessment and with LC and MG analysed final patient data. All authors read and approved the final manuscript.
